# Editorial: Psychoactive plants and fungi: biochemistry and genomics

**DOI:** 10.3389/fpls.2023.1327345

**Published:** 2023-11-08

**Authors:** Luis Alejandro de Haro, Paula Berman, Marcello Iriti

**Affiliations:** ^1^ Department of Plant and Environmental Sciences, Weizmann Institute of Science, Rehovot, Israel; ^2^ Department of Biomedical, Surgical and Dental Sciences, University of Milan, Milan, Italy

**Keywords:** psychoactive plants, biosynthetic pathways, metabolic engineering, gene editing, metabolomics

Psychedelic-assisted therapy is reshaping mental health treatment paradigms by harnessing the potential of psychoactive substances to address conditions like depression, post-traumatic stress disorder (PTSD), and addiction (Reiff et al., 2020). In this context, the historical use of nature-derived compounds throughout civilizations offers a robust foundation for their reevaluation and innovative applications. Amid this renaissance in psychedelic research, our Research Topic, “*Psychoactive Plants and Fungi: Biochemistry and Genomics*,” explores the intricate realm of these extraordinary organisms. The title seeks to encompass a diverse array of phylogenetically unrelated natural sources of psychoactive molecules, providing invaluable models for investigating the architecture and evolution of biosynthetic pathways of metabolites and their interactions with other organisms.

Cannabinoids have been one of the hottest topics of recent worldwide public health discussion. At the heart of these discussions is their therapeutic potential in alleviating a broad spectrum of health conditions (Kogan and Mechoulam, 2007). Rare cannabinoids, whether derived from *Cannabis sativa* (*Cannabis*) (Walsh et al., 2021), *Helichrysum umbraculigerum* (Berman et al., 2023), or synthetic biology strategies (Luo et al., 2019), play a pivotal role in developing tailored treatments for individual patients and specific illnesses. However, in the 21^st^ century, it is imperative to underscore that adopting novel crops or biotechnological production strategies must prioritize environmental considerations as a fundamental aspect of responsible innovation.

In this vein, the first work in our Research Topic by Seemakram et al. introduces us to the world of arbuscular mycorrhizal fungi (AMF), which act as symbiotic underground allies by developing extraradical mycelia that extend the capacity of *Cannabis* roots in the acquisition of nutrients of low mobility. This complements the application of fertilizer and boosts growth and cannabinoid content. Comparably, Welling et al. explore the effects of methyl jasmonate on cannabinoid production in *Cannabis*, unveiling a profound dose-response relationship and offering new potential genetic regulators by transcriptomics. Both works represent systematized knowledge that can be directly translated into superior production systems.

In combination with better practices, genetic improvement holds the promise of creating more efficient and environmentally friendly crops. The review article by Ingvardsen and Brinch-Pedersen overviews the challenges and possibilities of new breeding techniques in *Cannabis*, shedding light on the potential modulation of the plant’s chemical profile through genetic manipulation. In parallel with our capacity to edit plant genomes, it is imperative that we deepen our understanding of the genetic underpinnings of metabolic traits. Yuan et al. introduce a novel and high-resolution multiparent advanced generation inter-cross (MAGIC) population in tobacco, which facilitates precise genetic dissection and allows the identification of a novel transcription factor responsible for nicotine accumulation. This marks a significant advancement in our comprehension of polygenic metabolic traits.

Synthetic biology is an additional promising approach for the sustainable production of rare or unnatural psychoactive molecules. Monoterpene indole alkaloids, exemplified by harmine and harmaline found in the traditional Ayahuasca brew, possess monoamine oxidase-inhibiting properties and elicit diverse psychedelic experiences when consumed in combination with DMT (Brierley and Davidson, 2012). By introducing biosynthetic genes into *Nicotiana benthamiana*, the research by Boccia et al. creates novel analogs of alkaloids with new pharmacokinetic properties, offering a glimpse into the potential for engineering compounds with significant medical implications.

This Research Topic exemplifies the synergy between diverse research fields unified to understand better and improve the historical relationship between humans and psychoactive molecules. From symbiotic relationships to genetic improvement, from metabolic engineering to genome editing, this Research Topic provides a holistic view of the biotechnological potential of plant and fungi sciences in the age of renewed interest in psychedelics. We hope readers will find inspiration and knowledge alike within these pages to foster this vital exploration further.

## Author contributions

LdH: Writing – original draft. PB: Writing – review & editing. MI: Writing – review & editing.
